# Supplementary material for: An epifluorescence microscope design for naturalistic behavior and cellular activity in freely moving Caenorhabditis elegans
Source: Nat Commun. 2026 May 19;17:4411. doi: 10.1038/s41467-026-72709-w (PMC13187321; doi:10.1038/s41467-026-72709-w)
Supplement: Supplementary file 1 — Supplementary information [file 41467_2026_72709_MOESM1_ESM.pdf]

Build guide and parts list

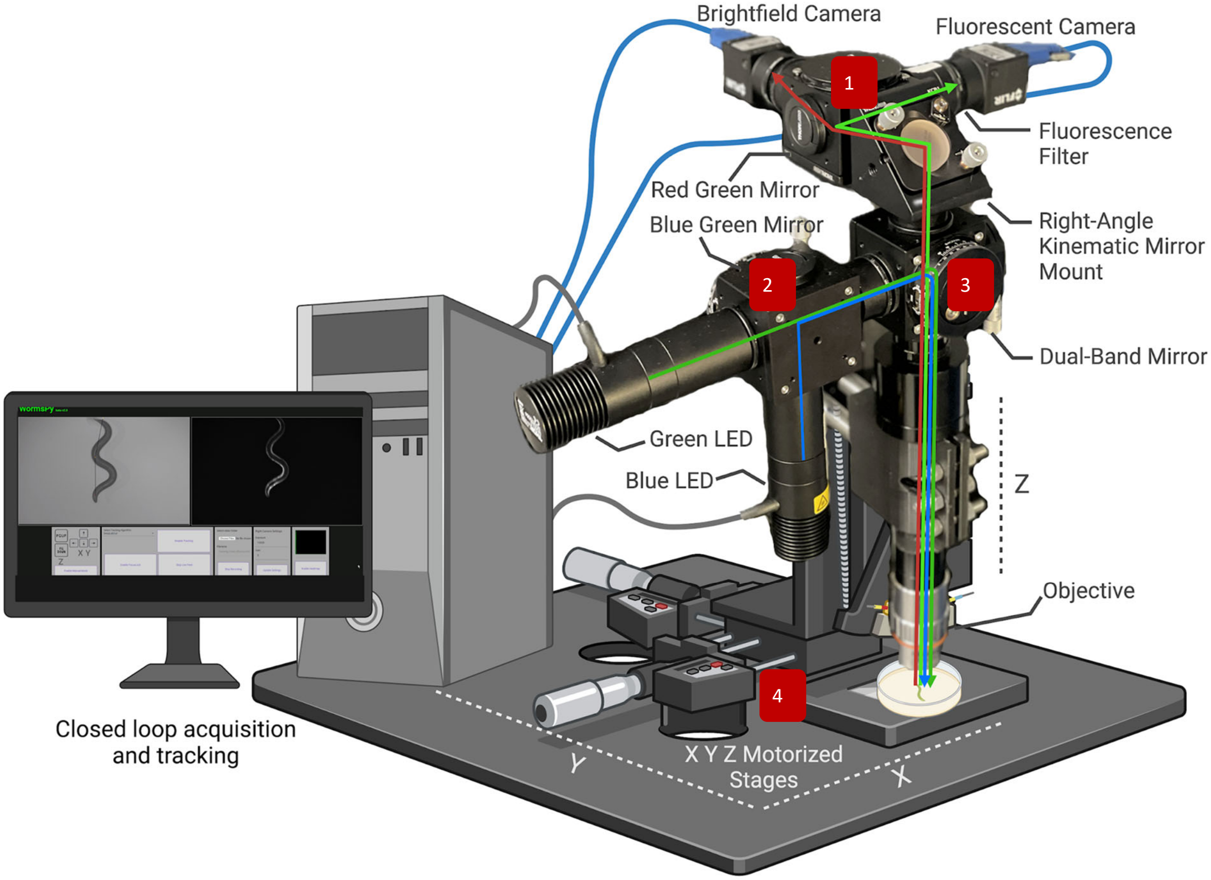

[#] refers to the index number of the component in the table below.
We recommend using C-mount compatible components for ease of assembly and to minimize converters. The core of the build consists of three Thorlabs 30mm cage cubes [1] with a blank cover plate [2] on one face and optical components or open ports on other faces. Mirrors are mounted inside the cage cubes using either precision rotation or fixed mounts [3,4,5]. Components are mechanically connected using fixed or adjustable C-mount extension tubes as needed [6]. This configuration is designed for ratiometric (green/red) calcium imaging at high magnification. The specific components listed here reflect that application, but the modular design allows straightforward substitution of cameras, optics, or illumination components for other imaging modalities.

1. Camera Cube: The imaging cube houses two computer-vision cameras [7] selected for high quantum efficiency to maximize sensitivity. Red and green emission filters [30,31] are mounted inside a short lens tube [8] immediately upstream of each camera. Emitted fluorescence is spectrally separated using a dichroic mirror [35] mounted in a fixed cage cube configuration [4,5], which was sufficient for camera alignment in our implementation. If finer angular adjustment is required, this element can be mounted on an additional precision rotation platform [3,5].

1. LED Cube: The LED excitation cube contains a blue and a green LED [11,12], each driven by a dedicated LED driver [13]. Light from each LED is diffused using an aspheric condenser lens [14] mounted in a lens tube [15] with a retaining ring [16]. A blue/green dichroic mirror [34], mounted on a precision rotation platform [3,5], combines both excitation wavelengths and directs them toward the central light-path cube.
2. Central Cube: The central light-path cube mechanically connects the LED cube and the camera cube using adjustable C-mount extension tubes [6]. It contains a dual-band mirror [36] mounted on a precision rotation platform [3,5], which directs excitation light toward the objective and emission light toward the imaging cube. The bottom face of the cube connects to a tube lens [17], a 100 mm lens tube [18], and an infinity-corrected objective [19]. In this infinity-corrected configuration, the tube lens defines the imaging magnification in combination with the objective.
3. The complete imaging assembly is mounted to the motorized stage using a 3D-printed clamp [22] (commercial alternatives are also available) that attaches directly to the 100 mm lens tube [18]. The clamp is supported by two optical posts [21], which connect to a right-angle mounting plate [20] affixed to the top of a vertical lift stage [23–26], providing Z-axis motion. The vertical lift stage is mounted on two TSB translation stages [27–29], which provide X- and Y-axis motion. Motors and cameras are connected to a PC via USB. Appropriate Zaber motor drivers and FLIR/Spinnaker camera drivers are required for compatibility with WormsPy.

Note: Fixed and adjustable C-mount extension tubes of various lengths were used as needed to mechanically connect components along the optical axis. In an infinity-corrected microscope, image magnification is determined primarily by the focal length of the tube lens (typically 180 mm or 200 mm standards), and by the distance between the tube lens and the camera sensor. Changing the effective tube-lens–to-sensor distance will alter the magnification on the camera. General guides to infinity-corrected optical systems are available on the Thorlabs YouTube channel.

Index	Name	ID	URL	Cost	#	Total (USD)
1	30 mm Cage Cube	C4W	https://www.thorlabs.com/thorproduct.cfm?partnumber=C4W#ad-image-4	69.01	3	207.03
2	Blank Cover Plate	B1C	https://www.thorlabs.com/thorproduct.cfm?partnumber=B1C	21.43	3	64.29
3	Precision Kinematic Rotation Platform	B4CRP/M	https://www.thorlabs.com/thorproduct.cfm?partnumber=B4CRP/M	336.58	2	673.16
4	Fixed Platform	B3C/M	https://www.thorlabs.com/thorproduct.cfm?partnumber=B3C/M	28.99	1	28.99

Index	Name	ID	URL	Cost	#	Total (USD)
5	Rectangular Filter Mount	FFM1	https://www.thorlabs.com/thorproduct.cfm?partnumber=FFM1#ad-image-0	66.68	3	200.04
6	Various C-Mount Extension Tubes as needed	CMV05, CMV10, etc;	https://www.thorlabs.us/navigation.cfm?guide_id=2416	36.54	8	292.32
7	Blackfly S Machine Vision USB Camera	BFS-U3-23S6M-C	https://www.flir.com/products/blackfly-usb3/?model=BFLY-U3-23S6M-C	595.00	2	1190
8	Lens Tube to hold filters	SM30L05	https://www.thorlabs.us/thorproduct.cfm?partnumber=SM30L05	32.67	2	65.34
9	Right-Angle Kinematic Mirror Mount	KCB1/M	https://www.thorlabs.com/thorproduct.cfm?partnumber=KCB1/M	154.65	1	154.65
10	Protected Silver Mirror	PF10-03-P01	https://www.thorlabs.com/thorproduct.cfm?partnumber=PF10-03-P01	56.26	1	56.26
11	Single-Color Cold Visible Mounted LEDs Blue	M470L5	https://www.thorlabs.com/thorproduct.cfm?partnumber=M470L5	244.11	1	244.11
12	Single-Color Cold Visible Mounted LEDs Green	M530L4	https://www.thorlabs.com/thorproduct.cfm?partnumber=M530L4	325.49	1	325.49
13	T-Cube™ LED Driver	LEDD1B	https://www.thorlabs.com/newgrouppage9.cfm?objectgroup_id=2616	348.22	2	696.44
14	Aspheric Condenser Lens	ACL2520U	https://www.thorlabs.com/thorproduct.cfm?partnumber=ACL2520U-A	33.19	2	66.38
15	SM30 Lens Tube	SM30L10	https://www.thorlabs.us/thorproduct.cfm?partnumber=SM30L10	35.05	2	70.1
16	Extra-Thick Threaded Retaining Ring	SM1RRC	https://www.thorlabs.us/thorproduct.cfm?partnumber=SM1RRC	12.24	2	24.48
17	Tube Lens	ITL 200	https://www.thorlabs.us/thorproduct.cfm?partnumber=ITL200	467.46	1	467.46

Index	Name	ID	URL	Cost	#	Total (USD)
18	100mm SM1 Lens Tube	35-761	https://www.edmundoptics.com/p/100mm-id1-lens-tube/33312	34.70	1	34.7
19	7.5X Mitutoyo Infinity Corrected Objective	66-383	https://www.edmundoptics.eu/p/75x-mitutoyo-plan-apo-infinity-corrected-long-wd-objective/20898/	1,420.00	1	1420
20	Right-Angle Mounting Plate	AP90/M	https://www.thorlabs.com/thorproduct.cfm?partnumber=AP90/M#ad-image-0	94.74	1	94.74
21	Optical Post	TR50V/M	https://www.thorlabs.com/thorproduct.cfm?partnumber=TR50V/M	15.46	2	30.92
22	3D-printed clamp (commercial alternatives available)	N/A	https://www.tinkercad.com/things/c5qEksqGSFS-tracking-scope-clamp?sharecode=OW20c2Icvekb8eVRaPaSOwbtwRGRAUTXbDLj7oibgIk	0	1	0
23	Vertical lift stage	VSR20A-T3A	https://www.zaber.com/products/vertical-stages/VSR	2,025.00	1	2025
24	12 VDC Power Supply	DS12	https://www.thorlabs.com/thorproduct.cfm?partnumber=DS12	60.97	1	60.97
25	X-MCC Series: Multi-axis universal motor controllers	X-MCC1	https://www.zaber.com/products/controllers-joysticks/X-MCC	995	1	995
26	Motor Extension Cable	MC10T3	https://www.zaber.com/products/accessories/MC10T3	30.00	1	30
27	TSB Series: Translation stages	TSB60E	https://www.zaber.com/products/linear-stages/TSB/specs?part=TSB60E	404.00	2	808
28	Micro linear actuators with built-in controllers	X-NA08A50-S	https://www.zaber.com/products/linear-actuators/X-NA/specs?part=X-NA08A50-S	1,313.00	2	2626
29	KX14A accessory kit	KX14A	https://www.zaber.com/products/linear-actuators/X-NA/specs?part=X-NA08A50-S	166.00	1	166
30	472nm Excitation Filter	67-027	https://www.edmundoptics.com/p/472nm-cwl-25mm-dia-30nm-bandwidth-od-6-fluorescence-filter/21567/	346.62	1	346.62

Index	Name	ID	URL	Cost	#	Total (USD)
31	575nm Excitation Filter	33-333	https://www.edmundoptics.com/p/575nm-cwl-25mm-dia-27nm-bandwidth-od-6-fluorescence-filter/2994/	346.62	1	346.62
32	520nm Emission Filter	67-030	https://www.edmundoptics.com/p/520nm-cwl-25mm-dia-36nm-bandwidth-od-6-fluorescence-filter/21570/	346.62	1	346.62
33	641nm Emission Filter	67-036	https://www.edmundoptics.com/p/641nm-cwl-25mm-dia-75nm-bandwidth-od-6-fluorescence-filter/21576/	346.62		346.62
34	Blue Dichroic Mirror	47-949	https://www.edmundoptics.com/p/50mm-square-45deg-blue-dichroic-filter/8155/	187.60	1	187.6
35	Red Dichroic Mirror	47-950	https://www.edmundoptics.com/p/50mm-square-45deg-green-dichroic-filter/8156/	187.60	1	187.6
36	Dual-band mirror	59022	https://www.chroma.com/products/sets/59022-et-egfp-mcherry-or-fitc-txred	1050.00	1	1050.00
	**TOTAL**					15929.55

Light Path Schematic

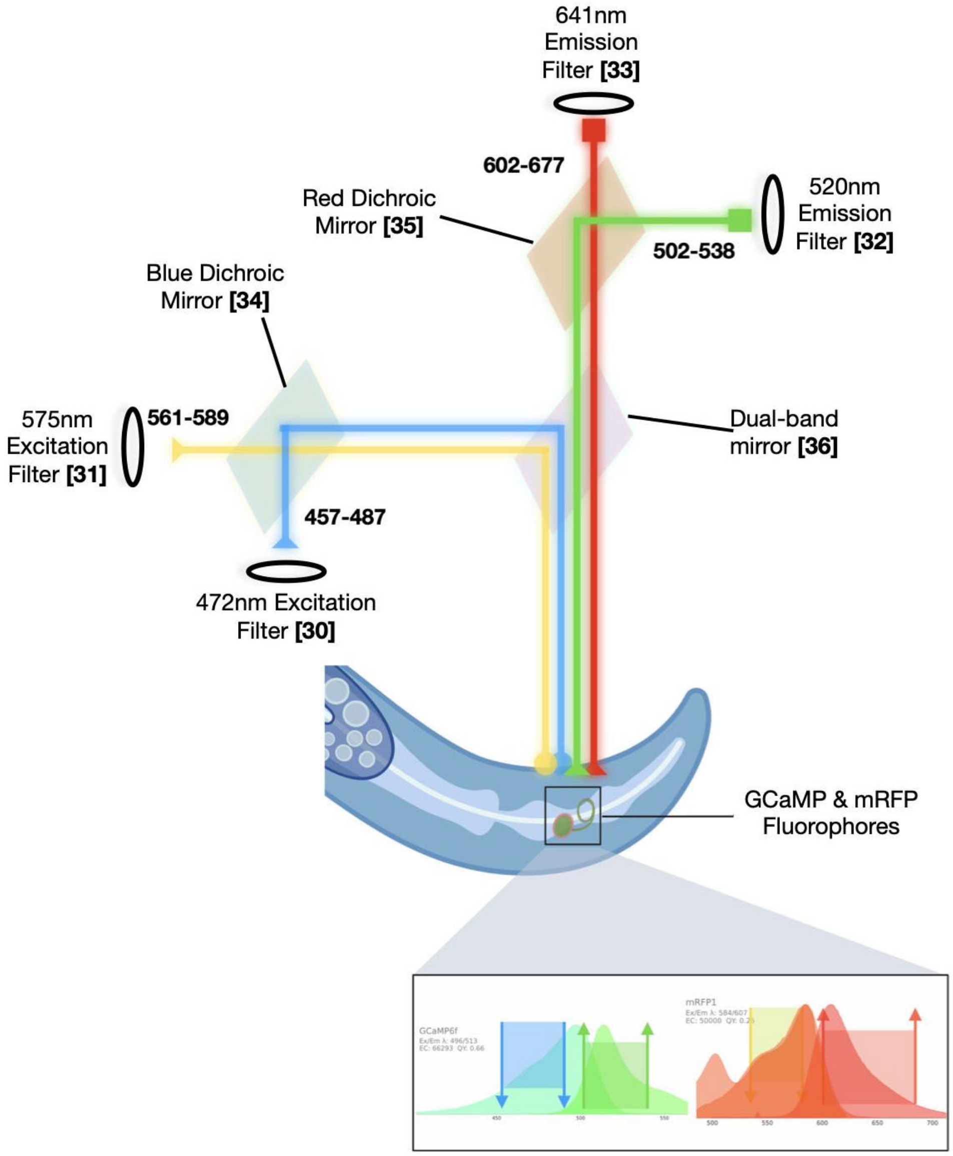

Supplementary Movie 1

User interface demonstration video.

https://youtu.be/mhyYDpziSE8

Supplementary Movie 2

Using Wormspy to record GCaMP7 expressed in *Drosophila* larvae muscle.

https://sebzdead.github.io/WormsPy/media/drosophila.gif
