# Supplementary material for: An epifluorescence microscope design for naturalistic behavior and cellular activity in freely moving Caenorhabditis elegans
Source: Nat Commun. 2026 May 19;17:4411. doi: 10.1038/s41467-026-72709-w (PMC13187321; doi:10.1038/s41467-026-72709-w)
Supplement: Supplementary file 3 — Description of additional supplementary files [file 41467_2026_72709_MOESM3_ESM.pdf]

Description of Additional Supplementary Files

Supplementary Movie 1

User interface demonstration video.

https://youtu.be/mhyYDpziSE8

Supplementary Movie 2

Using Wormspy to record GCaMP7 expressed in Drosophila larvae muscle.

https://sebzdead.github.io/WormsPy/media/drosophila.gif

Supplementary Data 1

Light microscopy reporting table
