# Supplementary figures and images for: An epifluorescence microscope design for naturalistic behavior and cellular activity in freely moving Caenorhabditis elegans
Source: Nat Commun. 2026 May 19;17:4411. doi: 10.1038/s41467-026-72709-w (PMC13187321; doi:10.1038/s41467-026-72709-w)

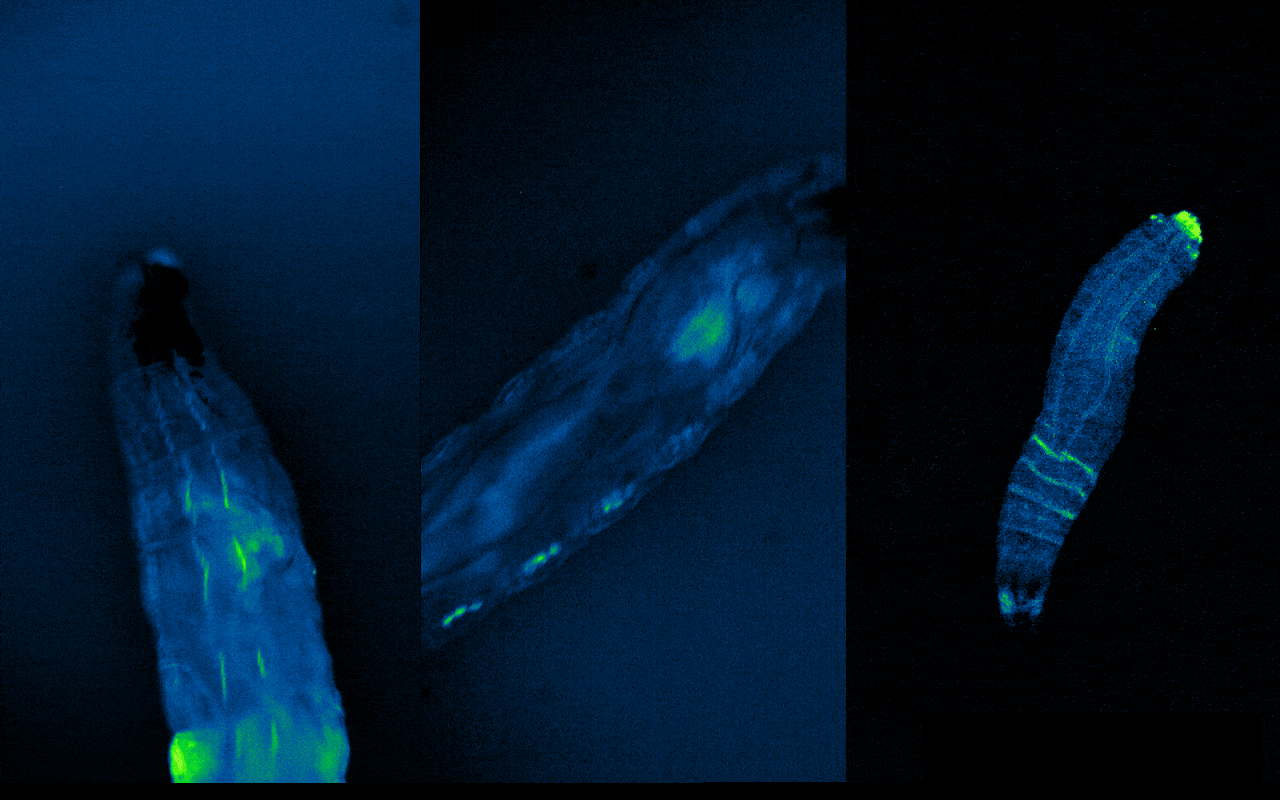

Supplement: Supplementary file 6 — Supplementary Movie 2 [file 41467_2026_72709_MOESM6_ESM.gif]

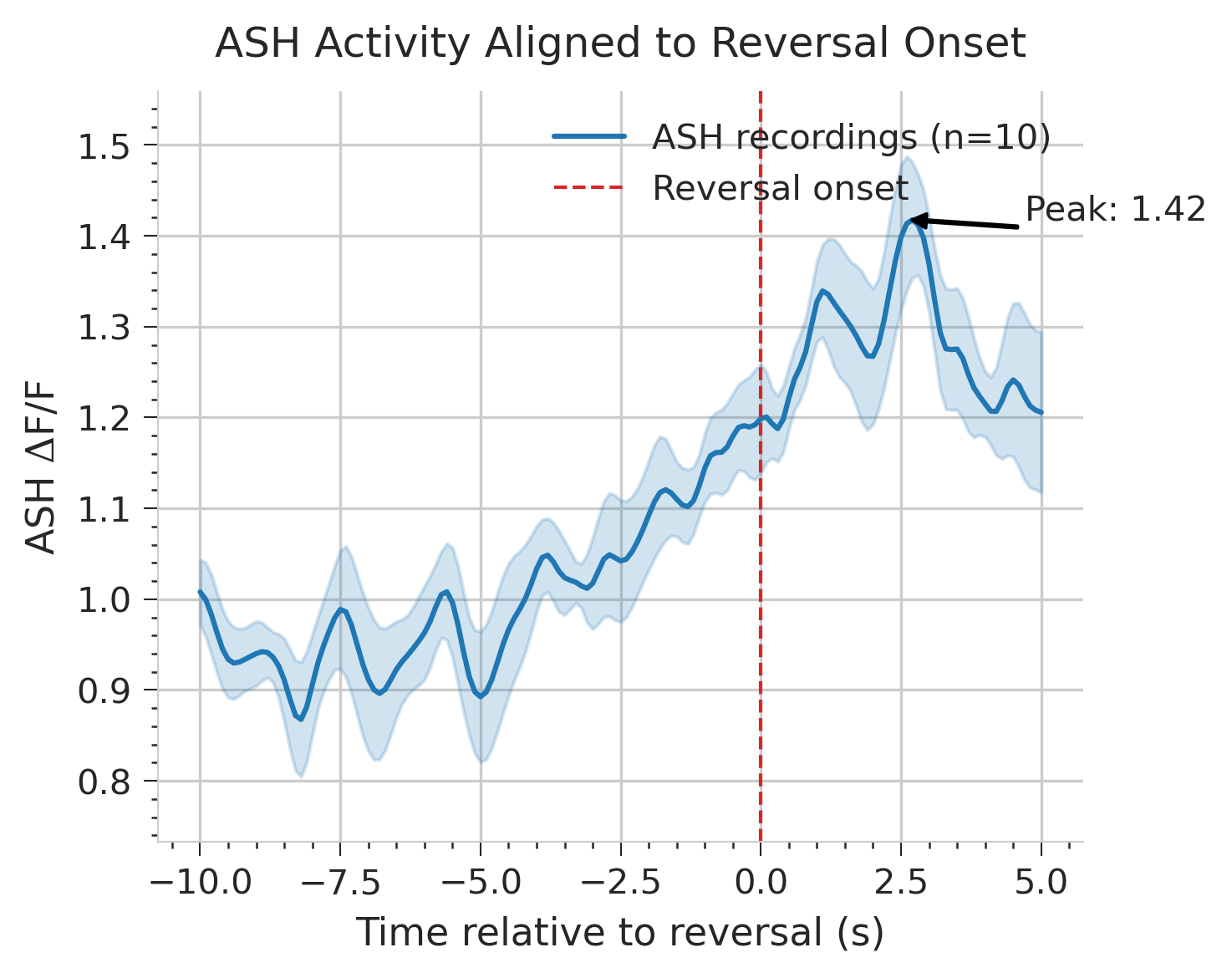

Supplement: Supplementary file 8 — Source data [file 41467_2026_72709_MOESM8_ESM.zip › fig3 ASH/TQ5856/ActivityTraces/ASH_reversal_activity.png]
